# RETRACTED ARTICLE: Practical application of wireless communication network multimedia courseware in college basketball teaching

**DOI:** 10.1186/s13638-021-01943-1

**Published:** 2021-04-06

**Authors:** Wengang Chen, Fang Wang

**Affiliations:** 1 Department of Physical Education, North Sichuan Medical College, Nanchong, 637000 Sichuan China; 2 Faculty of Arts, The University of Melbourne, Melbourne, VIC 3010 Australia

**Keywords:** Network multimedia, Basketball teaching, Empirical validity evaluation method, Multimedia courseware

## Abstract

With the acceleration of informatization and the coverage of wireless networks, homes, conferences, schools and other places have a higher pursuit of the wireless transmission capabilities of electronic devices. Wireless screen transmission technology is used more frequently in life, work and study. This article mainly discusses the practical application of network multimedia courseware in college basketball teaching. This article first elaborates the teaching plan of multimedia courseware, including teaching content, teacher guidance, student learning and multimedia courseware. Secondly, the multimedia courseware of basketball tactics basic teaching is completed by using Flash mx2004 plug-in. After that, it specifically introduces the process of how to transmit basketball teaching content through multimedia equipment to the video network for students to learn under the wireless network environment. It emphasizes that the “wireless multimedia communication” course is an important course in the electronic information subject. Finally, through the teaching experiment, the accuracy of the multimedia teaching method was tested, and the validity of the courseware content was tested by the empirical validity evaluation method. At the same time, after the teaching experiment, in order to test the two groups of students’ mastery of the basic coordination theory of basketball tactics, the basic coordination theory of basketball tactics was tested. The experimental group had 22 students with a score of 90 or more, accounting for 27.5%, and the control group had 13 students with a score of 90 or more, accounting for 16.5%. The results show that wireless network multimedia computer-assisted teaching has a positive effect on improving students’ interest in learning.

## Introduction

In the field of multimedia communication, various technologies are developing rapidly. Whether problems can be discovered and raised in time is related to whether they can take the initiative in this field. As a current technology hotspot, artificial intelligence is playing an increasingly important role in various industries and fields. The discovery, proposal and solution of many problems are inseparable from the development and progress of artificial intelligence technology. Therefore, in the artificial intelligence environment, we must learn to discover and ask questions from the perspective of artificial intelligence. The introduction of methods and ideas in the field of artificial intelligence into the "wireless multimedia communication" course can transform the classroom form into a problem-oriented approach, and cultivate students' ability to find and ask questions in an artificial intelligence environment.

Therefore, the main operations of the algorithms used in the wireless communication and multimedia fields are finally boiled down to the processing of matrices and vectors. At present, in order to pursue higher performance and efficiency, the above algorithms are widely used in a variety of different hardware environments. The abstraction of teachers' oral explanations, the limitations of demonstration actions and the complexity of tactics make technical movements and tactics teaching become the focus and difficulty of basketball teaching, and the emergence of basketball animation effectively solves the problem. Under the effect of network multimedia courseware teaching, the more difficult abstract thinking, language expression, logical thinking obstacles, etc., can be easily resolved, fully mobilizing students' enthusiasm for learning, guiding students' interest in thinking and exploring, and creating a learning atmosphere that is eager to learn.

Multimedia has affected many areas of education. Sidhu MS proposed an effective conceptual courseware development model, specifically for children with reading disabilities. He identified five basic functions to support the model, namely interaction, activity, background color customization, text reading (left and right) recognition and detailed description. He developed a prototype courseware based on the suggested model and tested it with a small group of children with dyslexia from selected schools in Malaysia. His research sample is too small [1]. In response to the growing importance of online education in social work education, Alston ST introduced the background of online teaching in social work education. In the context of the historic black university (HBCU), online teaching was further discussed. He emphasized the importance and necessity of well-trained teachers teaching in an online environment (especially in HBCU). He provided an example of how HBCU University of the District of Columbia prepares its faculty and staff to teach online, and use online teaching and social work. The teacher’s specific strategy ends. His research lacks comparative data [2]. Luo T believes that as more and more K-12 students study online in full-time online schools and blended learning environments, universities must prepare future educators for them in a virtual environment including clinical practice conduct teaching. Before engaging in online site placement, pre-service teachers must face online K-12 teaching. He used a design-based hybrid approach research method and sampled samples from four parts of the hybrid technology integration course. The method he proposed is not rigorous [3]. Rhode J believes that many institutions have adopted a universal method to develop online teaching teachers, which cannot meet the needs of teachers who often have different experience, skills and self-efficacy levels in online teaching. In order to solve these problems, he designed and implemented an online teaching readiness self-assessment. The tool is developed based on the key attributes and skills required for online teaching proficiency, covering three areas: online teaching experience and attitude, learning management system proficiency, and access to technology. The self-assessment was distributed through a web-based survey tool to teachers who were determined to develop new online courses. The individual results are used to create a personalized framework for professional development products (workshops, institutes, videos, and consulting) and provide timely resources to support the teacher's development process. His research has no practical value [4].

This research mainly discusses the practical application of network multimedia courseware in college basketball teaching. In the example of this research, FlashMX2004 is used to complete the multimedia courseware of basketball tactics basic teaching. The whole courseware is easy to embed in the webpage and suitable for transmission on the Internet. Use the scanner to scan the text part into the computer, recognize it with Hanwang HWOCR5.0 recognition software, and edit it in Word. Use the video processor and audio processor that comes with Kingsoft Hasee II to convert the VCD to the.avi video file format, use Windowsmoviemaker to edit and integrate the edited film, and finally import the required materials into Flash as needed to make the courseware. In order to ensure the correctness and effectiveness of the basic actions in the courseware, the empirical validity evaluation method is adopted, and the validity of the courseware content is tested.

## College basketball teaching

### Application of network multimedia courseware in physical education

The first is to break through the time and space limitations of conventional classrooms and create better teaching scenarios [5, 6]. Make full use of modern computer multimedia technology to create a teaching scene based on multiple sensory stimulations such as sound, image, animation, text, etc. Students understand the action structure in detail, form a complete action representation, and maximize the teaching effect [5]. The second is to reduce the complexity of theoretical knowledge to stimulate students' interest and achieve long-term memory effects [7, 8]. "Interest is the best teacher," multimedia computer can create a positive and happy emotion. Through the strong expressive power of the pictures, sounds and texts of multimedia courseware, students are actively willing to learn in this emotion [9]. Overcoming the boring content of books, turning some "dead" theoretical knowledge into "living," simplifying the complexity, increasing the interest of the knowledge, the students' attention is more concentrated, and the thinking is naturally active [10, 11]. The use of multimedia technology can optimize the physical education teaching process, impart high-density knowledge, break through teaching difficulties, and use the powerful graphics processing functions of computers to weaken or eliminate the learning obstacles of abstract thinking and language expression [12]. Sports theory knowledge can be made into vivid curriculum software, so that students can complete the mastery of knowledge even after class (zero class hours) [13, 14]. Increase the capacity of knowledge so that students can better master the content they have learned. Moreover, the expressive means of pictures, sounds and texts are more conducive to students' long-term memory of knowledge and receive teaching effects that cannot be matched by conventional classroom teaching [15].

### Basketball shooting

The knowledge of human movement development characteristics can help physical education teachers to understand the typical characteristics of students' movement skill development levels at various ages [16, 17]. For example, use the "whole sequence" to describe the general characteristics that appear on most students' bodies, or use the "partial sequence" to discover the special characteristics of a few students on the legs or other specific parts of the body, and then in physical education, according to The level or characteristics of its physical movement development determines what kind of physical education content to choose, and then the improvement of human motor skills changes with age and the level of physical education [18].

1. Assuming that the basketball touches the backboard or the size of the basket after the ball is shot [19, 20]. Therefore, according to Newton's law, the equation at time t is:
  1 $$V_{x} = V_{1} * \cos \theta$$
  2 $$V_{y} = V_{1} * \cos \left( { - gt} \right)$$

Among them, $g$ is the acceleration of gravity [21]. From this, the trajectory of the center of the ball can be obtained as the following parabola:

3 $$y = x\tan \theta - x^{2} \frac{g}{{2v_{1}^{2} \cos^{2} \theta }}$$

Let $x = L$,$y = H - h$ get the condition that the ball hits the center of the basket:

4 $$\tan \theta = \frac{{v_{1}^{2} }}{gL}\left[ {1 \pm \sqrt {1 - \frac{2g}{{v_{1} }}\left( {H - h + \frac{{gL^{2} }}{{2v_{1}^{2} }}} \right)} } \right]$$

In addition:

5 $$1 - \frac{2g}{{v_{1}^{2} }}\left( {H - h + \frac{{gL^{2} }}{{2v_{1} }}} \right) \ge 0$$

It can be solved for $v_{1}$:

6 $$v_{1}^{2} \ge g\left[ {H - h + \sqrt {L^{2} + \left( {H - h} \right)^{2} } } \right]$$

It can be seen that $v_{\min }$ is the decreasing function of $h$ [22, 23]. The incident angle $\psi$ when the ball enters the basket can be obtained from the following formula:

7 $$\tan \psi = \tan \theta - \frac{{2\left( {H - h} \right)}}{L}$$

Through the above analysis, we can get the relationship between shooting angle, speed and basket angle of different shooting heights in case 1 [24].

(2) Suppose that the basketball hitting the backboard is not considered after the hand is obtained, but the size of the basket is considered. Suppose the diameter d of the basketball and the diameter D of the basket.
  8 $$\sin \psi > \frac{d}{D}$$

When the ball enters the frame, the center of the ball can deviate from the center of the frame, and the maximum distance forward is $\Delta x$, then:

9 $$\Delta x = \frac{D}{2} - \frac{d}{2\sin \psi }$$

Make $y = H - h$, you can get:

10 $$x^{2} \frac{g}{{2v_{1}^{2} }} - x\tan + H - h = 0$$

Ask for guidance on $\theta$ and make $x = L$, then:

11 $$\frac{{{\text{d}}x}}{{{\text{d}}\theta }}\left| {_{x = L} } \right. = \frac{{L\left( {v_{1}^{2} - gL\tan \theta } \right)}}{{gL - v_{1}^{2} \sin \theta \cos \theta }}$$

Replace the derivative on the left with $\Delta x/\Delta \theta$ approximately to get the following relationship between the deviation of the shot angle $\Delta x$ and $\Delta \theta$:

12 $$\Delta \theta = \frac{{gL - v_{1}^{2} \sin \theta \cos \theta }}{{L\left( {v_{1}^{2} - gL\tan \theta } \right)}}\Delta x$$

Similarly, seek guidance on $v_{1}$ and make $x = L$, then:

13 $$\Delta v = \frac{{gL - v_{1}^{2} \sin \theta \cos \theta }}{{gL^{2} }}v_{1} \Delta x$$

The relative deviation of $v_{1}$ can be obtained as:

14 $$\left| {\frac{\Delta x}{{v_{1} }}} \right| = \left| {\Delta \theta \left( {\frac{{v^{2} }}{gL} - \tan \theta } \right)} \right|$$

In this case, the pitch angle $\theta$ can be obtained as:

15 $$\tan \theta_{\min } = \frac{{v_{1}^{2} }}{gL}$$

### Courseware design

**Fig. 1:**
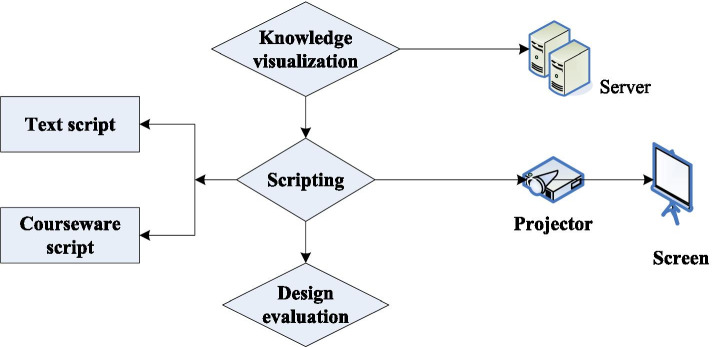
Courseware design process

Making a multimedia courseware needs to start from the textbook and instructional design, collect and create all kinds of materials needed in it, and then make and debug and run [25]. It is especially necessary for an ordinary teacher to understand and master the methods of software engineering. According to the life cycle method of software engineering, the whole system consists of 5 parts. The courseware design process is shown in Fig. 1.

1. Overall scheme design
  Before developing a multimedia courseware, we should first analyze the overall needs of the courseware, the goals achieved by the courseware and the material conditions needed to make the courseware, determine the development time, testing and actual application time, which is called demand analysis. After that, the overall plan design of the courseware was made. The purpose of the overall plan design is to determine whether the educational thought can be realized with the corresponding computer technology [26].
2. Script design of courseware
  After in-depth study of the syllabus and clarification of the teaching content, the curriculum is divided into several knowledge points. Select the development tool, the media material format used for each knowledge, and write the result of the courseware design into a courseware script.
3. Courseware production

After the script is designed, start to make the courseware, the steps are as follows:

1. Select and design materials.
2. Make multimedia courseware.
3. Online publication of multimedia courseware.
4. Test evaluation.
  Courseware evaluation basically proposes the basic content and main indicators of the courseware from the aspects of information presentation, human–computer interaction, teaching process control, and document provision.
5. Promotion and application.

The promotion and use of courseware is very important. Unused or small-scale use of courseware that consumes a lot of manpower development is undoubtedly a huge waste of manpower and financial resources. Therefore, a well-made courseware should be promoted and issued in time Let more people benefit. Therefore, before the courseware is developed, the preliminary document design of the courseware must be done well. During the courseware development period, we must spend more time for mid-term production. In the later stage of courseware development, the operation, testing and modification are repeated cycles of analysis, design, development, and operation.

### Wireless communication

With the rapid development of the information industry, the integration between remote wireless transmission technology and computer technology is bound to be a trend. The popularity of the Internet has accelerated the integration of these two technologies, and the rapid development of mobile communications has proposed wireless remote transmission higher requirement. Wireless remote transmission can transmit various forms of information such as image, audio and video. Its characteristics are high speed, multimedia support, and multiple service channels. In terms of transmission speed, it is no less than the traditional data transmission method, and it can also expand other businesses. Compared with traditional data transmission methods, remote wireless data transmission has broader application prospects. With the advent of the 5G era, it will surely be able to provide users with efficient transmission speeds and high-quality multimedia services.

1. Bluetooth technology
  Bluetooth technology is a protocol wireless network technology, which enables various communication devices and even household appliances to connect wirelessly. But because of its frequency hopping technology, its battery life can only last a few days. In addition, the scalability of Bluetooth is not very good, and a network can only support a few devices at most.
2. WIFI technology
  The communication of WIFI technology is more convenient, its transmission rate is faster than other technologies, and its advantages are more obvious. However, its battery usage is not optimistic, and some will even run out within a few hours, which cannot meet our requirements for low power consumption and low cost.
3. Wireless USB technology
  Wireless USB technology is developed based on UWB. It is different from WIFI and Bluetooth, it is a kind of non-carrier communication. It has the characteristics of low cost and long battery life, but it is not suitable for long-distance transmission, and it also lacks in safety and scalability.
4. Zigbee technology
  Zigbee technology is a communication technology with high reliability and low power consumption. However, its transmission rate is relatively slow and the transmission distance is relatively short, so it is not suitable for long-distance transmission.
5. NFC technology
  NFC technology is the product of the modification and integration of interconnection technology and radio frequency identification technology. Its operating frequency is 13.56 MHz. Compared with several other wireless communication technologies, its reliable transmission distance is shorter, but its speed and stability are better than infrared.
6. UWB

UWB is also known as impulse radio, which communicates in impulse mode. Although this technology has strong anti-interference performance and has great advantages in bandwidth, power consumption, and safety performance, it is inconvenient to use in long-distance applications due to the limitation of transmission distance.

Through the comparison of these technologies, it is not difficult to find that the goal of this system is to achieve the requirements of long-distance, high-speed wireless transmission in large classrooms, large-scale support equipment, and ultra-low power consumption power-saving modes. This technology is far from meeting the requirements of our goals. Therefore, we found that the wireless communication technology implemented with nRF24Le1 chip is the most suitable for this interactive teaching system.

## College basketball teaching experiment

### Operating environment of courseware

1. Hardware operating environment: CPU Pentium II, RAM 64 MB, graphics card true color (16-bit), 16-speed optical drive, sound card, 4G or more hard disk space.
2. Software operating environment: operating system Windows98/2000/XP, Flash7.0 plug-in.
3. Courseware development software: Photoshop6.0, Kingsoft Kingsoft II, ACDSee7.0, MicrosoftWordXP, FlashMX2004.
4. Courseware development hardware: CPU Pentium IV, memory 256 MB, graphics card true color (16-bit), DVD drive 16-speed, sound card, 40G hard disk space, etc.

### System design

1. The specific formulation of teaching objectives. According to demand analysis, the predetermined teaching objectives are refined. According to the attributes of each knowledge point (facts, concepts, principles, technical characteristics, problem solving, etc.), list the levels.
2. The choice of teaching strategies. According to the teaching objectives of the course, use this courseware as a part of auxiliary teaching.
3. The choice of courseware media. According to the content of the textbook, first write the teaching plan of "Basketball Tactics Basic Coordination," then consider the characteristics of multimedia, reprocess, and write the teaching plan of multimedia courseware.
4. The teaching plan of "Basketball Tactics Basic Cooperation" usually includes teaching objectives, tasks and requirements, teaching key points, teaching difficulties, class time arrangement, teaching method and learning method, ending part, exercise load estimation, venue equipment, and after-school summary.
5. The multimedia courseware teaching plan of "Basketball Tactics Basic Cooperation" The multimedia courseware teaching plan consists of at least four parts: teaching content, teacher guidance, student learning and multimedia courseware. Multimedia courseware is only a part of the whole teaching process, and the interaction and interaction of these four parts should be fully considered.

### Teaching plan of multimedia courseware

1. Introduction
  The teacher points out the key points and difficulties of this course, students have a preliminary understanding of this course, and the multimedia courseware displays the entire content catalog of this course.
2. Each chapter
  The teacher explains and demonstrates the essentials of the action, which is convenient for students to learn and understand. The multimedia courseware fully displays all the teaching content in the form of pictures, texts, and animations.
3. Video analysis
  Teachers decompose actions according to video explanations, students discuss and explore the characteristics of tactical basic coordination, and multimedia courseware controls the playback of videos by playing, pausing, turning forward, turning back, etc., which is convenient for analysis and explanation.
4. Test questions

Teachers provide test questions and students conduct test questions to consolidate the mastery of knowledge points. Multimedia courseware is controlled by programs to automatically change questions, calculate scores, and give standard answers.

### Courseware production

1. Selection of courseware making tools
  In the example of this research, FlashMX2004 is used to complete the multimedia courseware of basketball tactics basic teaching. The whole courseware is easy to be embedded in the web page and suitable for transmission on the Internet. Use the scanner to scan the text part into the computer, recognize it with Hanwang HWOCR5.0 recognition software, and edit it in Word. Use the video processor and audio processor of Kingsoft Hasee II to convert the VCD to the.avi video file format, use Windowsmoviemaker to edit and integrate the edited film, and finally import the required materials into Flash as needed to make the courseware.
2. The validity of the courseware
  In order to ensure the correctness and effectiveness of the basic actions in the courseware, the validity of the content of the courseware was tested and the empirical validity evaluation method was adopted. Ten experienced teachers (including 4 professors and 6 associate professors) are invited to review the initial courseware, and evaluate the courseware at level four, namely, accuracy, basic accuracy, suggested revisions and inaccurate evaluations, based on the opinions and suggestions of experts, to modify and supplement the courseware. The feedback of such survey opinions is an iterative process, and the audit results are shown in Table 1.
3. Data collection of courseware

**Table 1:** Audit results

Conclusion	Accurate	Basically accurate	Suggest modification	Inaccurate
Number of people1	3	5	2	0
Number of people2	5	4	1	0

1. Image source:
  ① After the image is obtained by the digital video camera, it is directly input to the computer.
  ② Using Super Jieba 2000 to directly intercept relevant VCD disks and store them in the computer.
  ③ Download relevant images from other web pages and save them to the computer.
2. Image source:
  ① After the image is obtained by the digital camera, it is directly input to the computer.
  ② Use the scanner to scan the relevant pictures and input them into the computer.
  ③ Download relevant photographs from other webpages and save them to the computer.
3. Sound effect source:
  ① Use the computer program-attachment-recorder to record the sound, and save it in the computer in the form of WAV file.
  ② Intercept the sound files in other software, obtain the files (WAV and MID) and save them in the computer.
  ③ Download relevant sounds from other web pages and save them to the computer.
4. Wireless transceiver module.
  Adopt nRF24Le1 wireless radio frequency module, set reasonable transmission rate and signal transmission modulation mode, realize high-speed, high-quality, low-power, long-distance wireless transceiver communication of information.
5. Text source:

On the Founder Aos development platform, use Word to write directly.

The PLC remote wireless communication system mainly completes two parts of work: PLC data remote collection and program remote maintenance. The realization of these two functions is completed on the basis of network communication. The following will combine the network communication function and the remote data collection function with the remote maintenance function of the program is tested.

Whether it is the communication between the wireless data transmission module and the server or the communication between the server and the client, it is necessary to follow a certain communication protocol to accurately complete the data exchange. The server must not only communicate with the PLC and the client, but the client must also communicate with the programming software. These are two different communication methods. The appropriate communication method should be selected according to their respective communication characteristics.

### Test content

Before the experiment, the physical fitness and basketball foundation of the experimental group and the control group were tested. The test content and standards are:

1. Basic test: full-court turnback (s) (time counting).
2. Basic technical test: 1 min shooting (counting the number), dribbling around three markers and layup (single-stroke basketball test content) (timing).
3. Basic tactics test: Three half-court passes, scoring, and screen tactics are used to test the success rate (10 attacks per group) (counting the number of goals).

## College basketball teaching

### Comparative results of the experimental group and the control group in the evaluation of students' theoretical knowledge

**Fig. 2:**
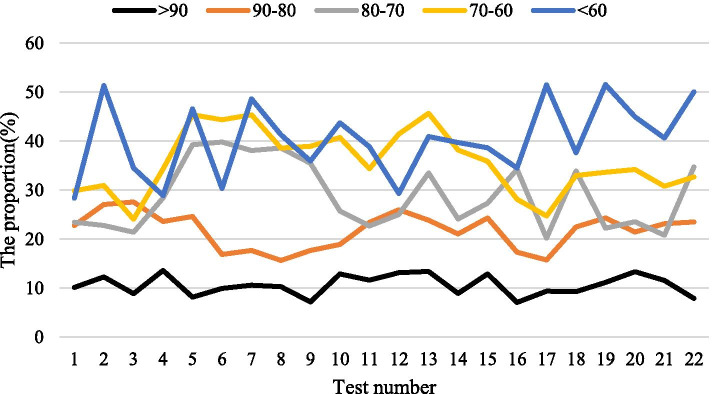
The ratio of the number of people in each segment of the test group

**Fig. 3:**
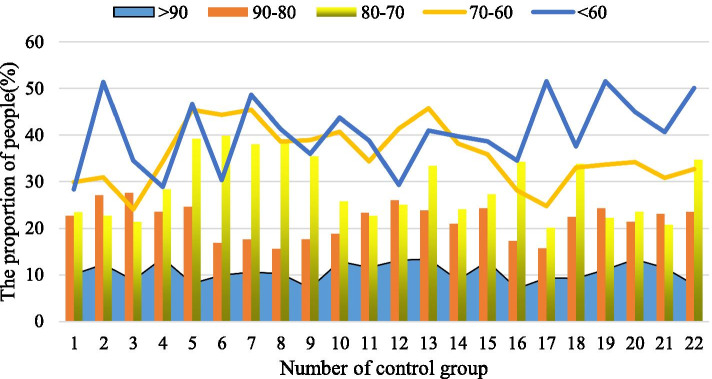
The ratio of the number of people in each segment of the control group

The way of PLC data collection through the wireless communication network does not need to lay a special cable for data transmission, only a computer with Internet access and client software installed on the computer can complete the reading of PLC data anytime and anywhere. Compared with the serial port method, this data collection method has great advantages in real-time and convenience. After the teaching experiment, in order to test the mastery of the basic basketball tactics and theoretical knowledge of the two groups of students, a test of basic basketball tactics and theoretical knowledge was carried out. The test questions were mainly judgment and selection. The comparison results of test scores are shown in Table 2. The ratio of the number of people in each segment of the test group is shown in Fig. 2, and the ratio of the number of people in each segment of the control group is shown in Fig. 3. From the comparison results, 22 people with a score of 90 or more in the experimental group accounted for 27.5%, and 13 people with a score of 90 or more in the control group accounted for 16.5%. The students in the experimental group have a higher level of basketball theoretical knowledge than the students in the control group. The test result *P* < 0.01, the degree of difference is more obvious. In addition, in terms of curriculum content design, 82.19% think that the curriculum content design is appropriate; 69.43% think that the resources provided are very rich, and 12.08% think that they are not rich enough. 35.9% think that the self-test questions and practice questions after the chapter are troublesome or boring, so sometimes they do a little or basically not, so we have to work harder here. 69.81% think that the visual design of the webpage is good, which is in line with their spiritual pursuit. 89.21% think the course navigation design is better. 23.29% thought that there was no professional learning atmosphere in the learning forum, and the problem could not be solved in time, 34.12% thought it was a bit helpful, and 10.21% thought there was no topic to communicate. It can be considered that the basketball multimedia courseware can obviously promote the students to master the theoretical knowledge of basketball tactics.

**Table 2:** Test results comparison results

Group	*N*	> 90	90–80	80–70	70–60	< 60	(*X* ± *S*)	*T*	*P*
Test group	*N* = 80	22	51	5	2	0	87.2 ± 8.3	3.7	< 0.01
	Percentage	27.5	63. 75	6.25	2.5	0			
Control group	*N* = 80	13	48	12	6	1	82.1 ± 8.9		
	Percentage	16.5	60	15	7.5	1.25

### Satisfaction degree of the students in the experimental group and the control group

**Fig. 4:**
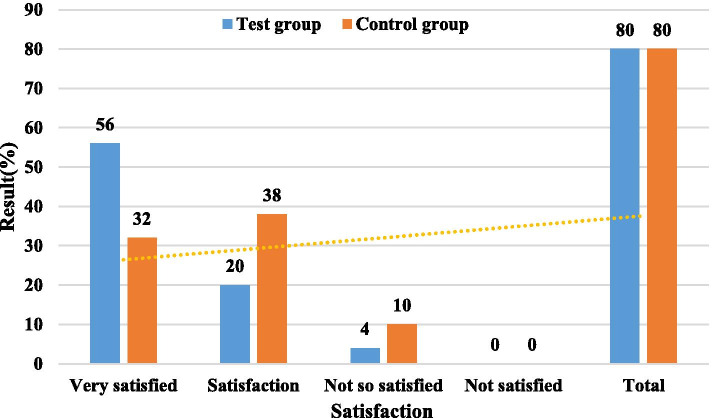
Satisfaction results of experimental group and control group

According to the system architecture of the Internet of Things technology, a large amount of information in distributed sensors usually flows into adjacent communication nodes, which are responsible for long-distance data transmission. Utilizing the advantages of the nano-generator itself to realize short-distance and fully self-powered wireless transmission, which can greatly reduce the overall power consumption of the communication system. In order to understand the degree of students' satisfaction with basketball teaching, the form of after-school questionnaire is used to solicit the opinions of the experimental group and the control group, as shown in Table 3. Statistics show that 95% and 87.5% of the students in the control group and the experimental group are very satisfied and satisfied with the basketball class. The satisfaction results of the experimental group and the control group are shown in Fig. 4. In the discussion, some students thought that the courseware was very helpful for learning and helpful for cultivating cognitive ability. Consolidate knowledge through exercises, and lay a solid foundation for future study and work, and I sincerely hope to use this kind of courseware to learn more in the future. Expert judges also agreed that the courseware was designed very well. It can be seen that multimedia computer-assisted teaching plays a positive role in cultivating students' interest in learning.

**Table 3:** Soliciting opinions from students in the experimental group and the control group

Group	*N*	Very satisfied	Satisfaction	Not so satisfied	Not satisfied	Total
Test group	*N* = 80	56	20	4	0	80
	Percentage	70	25	6	0	100
Control group	*N* = 80	32	38	10	0	80
	Percentage	40	47.5	12.5	0	100

### Comparative results between the experimental group and the control group

**Fig. 5:**
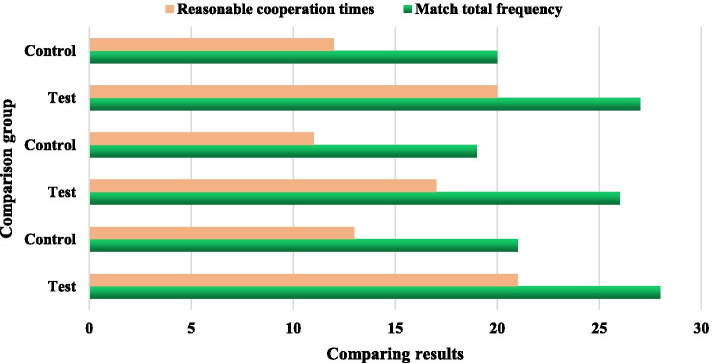
Comparison results between groups

In recent years, computer science and communication technology have been continuously developed, and have brought earth-shaking changes to our lives. At the same time, our requirements for the quality of life have also increased. In mobile communications, people began to pay more attention to the security and smoothness of wireless networks; in multimedia, people more hope that the pictures they see are clear and brilliant. This puts forward higher requirements for the application of computer science in this field, and also brings challenges to computer researchers who develop projects in this field. In order to test the effect of the implementation of the courseware, through the three-week teaching competition, after the teaching experiments of different groups are completed, 3 experienced teachers (1 professor and 2 associate professors) will conduct teaching competitions on their own teaching groups. The experiment results of the teaching competition are shown in Table 4. The comparison results between groups are shown in Fig. 5. It can be seen from Table 4 that the proportion of reasonable tactical cooperation times in the experimental group of group A is 75%, and the proportion of the control group is 61%; the proportion of reasonable tactical cooperation times in the experimental group of group B is 65%, and the proportion of the control group is 60%; The proportion of reasonable tactical cooperation in group C was 71%, and the proportion of control group was 58%. The comparison results show that the total frequency of cooperation and the number of reasonable cooperation in the basketball teaching game of the experimental group students are higher than those of the control group, and their mastery of basic basketball tactics is significantly higher than that of the control group students, and the degree of difference reaches the expected level. Therefore, we should use a variety of methods to comprehensively evaluate the learning effect of online courseware learners, or evaluate and analyze based on a standard index system. However, in this courseware, the evaluation is mainly based on expert evaluation. There is no mature idea for the evaluation and dynamic monitoring of those ill-formed knowledge, which needs to be further strengthened.

**Table 4:** Experimental results of teaching competition

Group	Group	Match total frequency	Reasonable cooperation times	%
Group A	Test	28	21	75
	Control	21	13	61
Group B	Test	26	17	65
	Control	19	11	60
Group C	Test	27	20	71
	Control	20	12	58

### Basketball tactics courseware in teaching by experimental group students

**Fig. 6:**
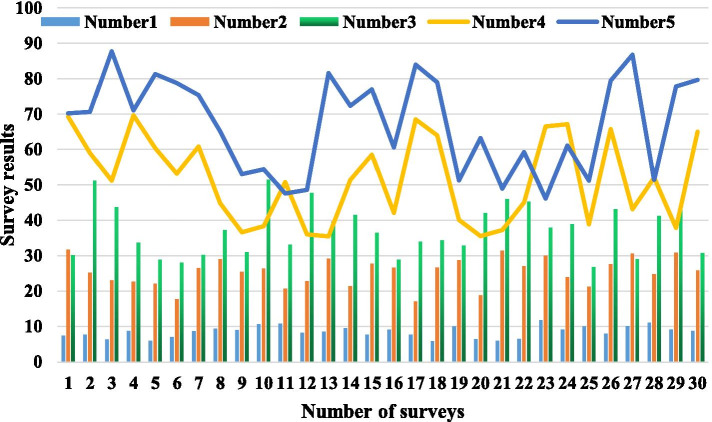
Survey and analysis summary results

The short-distance wireless transmission technology includes Bluetooth technology, Wi-Fi networking technology, ZigBee networking technology and other fields. Network interaction technology follows a wide range of protocols, each with its own advantages. Sensor technology is relatively mature, and the selection and use of sensors are also different. Long-distance mobile device communication technology mainly provides users with a human–computer interaction interface on the mobile phone. The implementation methods include Web development technology and Android development technology. In order to fully understand the effect of courseware teaching, a questionnaire survey is used to understand the evaluation of the experimental group students on the application of computer-assisted teaching methods to basketball tactics basic teaching. The results of the evaluation investigation and analysis are shown in Table 5, and the summary results of the corresponding investigation and analysis are shown in Fig. 6. The five items of the survey showed that the positive attitudes were 92.5%, 100%, 98.8%, 95%, and 91%; the general attitudes were 7.5%, 0, 1.2%, 5%, and 8.7%. This fully shows that students have a positive attitude towards using multimedia basketball courseware. Therefore, in conventional teaching, teachers' explanations and demonstrations are easily restricted by many factors such as time and space, and it is difficult to synchronize the two. Abundant and effective learning resources are a prerequisite for cultivating learners' cognitive ability and promoting meaningful learning in online learning. The learning resources in the network environment include course resources and extended resources. Curriculum resources are the main resources for courseware learning. The design should be based on learners' cognitive laws, combined with multimedia learning theory and contemporary cognitive theories. Extended resources are resources for learners to actively explore knowledge and provide support services for learners through network communication technology.

**Table 5:** Evaluation survey analysis results

Serial number	Investigate subject	Yes		General		No	
		*N*	Proportion	*N*	Proportion	*N*	Proportion
1	Is it useful?	74	92.5	6	7.5	0	0
2	Improve learning motivation?	80	100	0	0	0	0
3	Cultivate interest in learning?	79	98.8	1	1.2	0	0
4	Speed up content mastery?	76	95	4	5	0	0
5	Improve analytical capabilities?	73	91	7	8.7	0	0

**Fig. 7:**
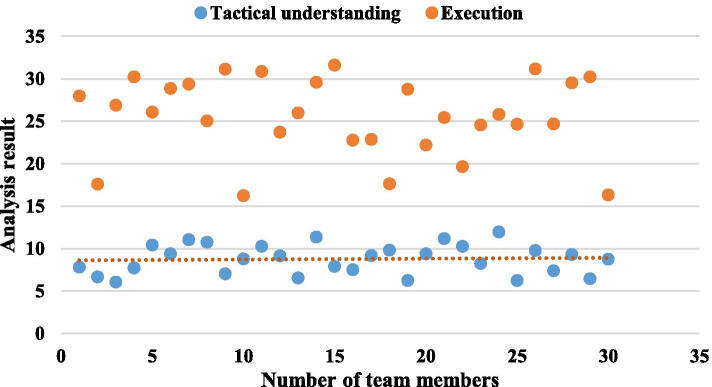
Tactical understanding and execution analysis results

Visualized action examples are more conducive to students' mastery of the content. Therefore, if you use multimedia technology, the teaching process is much simpler. Combining the teacher's explanation and guidance with the flash animation action video function demonstration, through the combination of the advantages of video technology in the sensory audiovisual and slow playback, pause, etc. Enable animation teaching to break through difficulties and strengthen key points. Another feature of animation is that it is convenient and efficient. It can break through the limitations of time and space, shorten the process of a certain object, or understand the relationship between its causes and results, so that it can highlight key points, disperse difficulties, and achieve obvious results. The limitations of teacher's action demonstration will inevitably cause errors in students' understanding. Using basketball animation to express these changes has the advantages of good time and space, strong overall effect, and deep impression. In the courseware, multimedia technology is used to intuitively express the basketball movement circuit, which is easy to understand and simplifies complex tactics. The results of tactical understanding and execution are shown in Table 6. From the comparison of the overall tactical understanding and execution effect of the experimental group and the control group, it is found that after 52 h of teaching, the test group's performance is significantly higher than the control group, and the difference between the two groups has reached a significant level of difference, indicating that the experimental group The teaching has a significant effect on cultivating students' tactical awareness and improving tactical judgment and application ability. The results of tactical understanding and execution analysis are shown in Fig. 7. The results of several test items show that there is no significant difference between the two groups after the test. This shows that the courseware method is no different from traditional teaching methods in technical mastery, but the difference between the two groups in tactical application and theoretical testing is very significant. The students in the experimental group have a significantly higher understanding of the entire tactics than the control group. They also have a solid and firm grasp of the theory teaching film after learning. It shows that in terms of theoretical teaching, it is superior to traditional coach oral explanation methods. In the interviews with students, most students hold a positive attitude towards the courseware method.

**Table 6:** Tactical understanding and execution effect results

Content	Test		Control	
	Before experiment	After test	Before experiment	After test
Technical compliance	27.8 ± 6.3	48.1 ± 7.2	27.3 ± 6.0	46.2 ± 6.1
Tactics	11.7 ± 14.3	56.2 ± 3.6	11.7 ± 4.2	21.9 ± 3.0
Overall result	79.1 ± 15.4	151.2 ± 17	79.1 ± 16	143.5 ± 18.1

### Effect of offenses

**Fig. 8:**
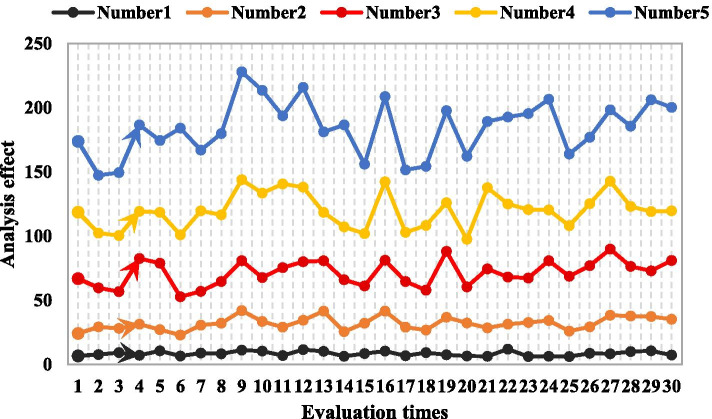
Analysis effect of offenses

In recent years, with the development of modern electronic technology, short-range wireless communication technology has begun to develop rapidly due to its low cost, which is conducive to a large number of promotion in the market, low power consumption and peer-to-peer communication. After the multimedia courseware teaching experiment is over, two experimental results are measured. The first is the theoretical and penalty test scores of the two groups of students who participated in the multi-media courseware learning experiment of intrusion and foul play. The second is the test results of the students' study attitude survey. After the teaching experiment, in order to test the two groups of students' learning and mastery of intrusion and foul content, the students were tested on the theoretical knowledge score and the video intrusion foul judgment score. The test questions were mainly judgment questions and multiple choice questions, and the intrusion foul test results. As shown in Table 7. The test scores are compared, and the comparison results between groups are obtained. From the statistical results, it can be seen that 11 people in the experimental group are excellent (above 90 points), accounting for 27.5%, and 7 people in the control group are excellent (above 90 points), accounting for 17.5%. The average scores of the three items of foul judgment in the experimental group were significantly higher than those in the control group. The theoretical test scores of the experimental group and the control group were tested for differences. Using single-factor analysis of variance, the P values were all less than 0.01, indicating that the differences are highly significant. The analysis effect of offenses is shown in Fig. 8. The accuracy of the students in the experimental group on the theoretical knowledge of intrusive fouls and fouls was significantly higher than the students in the control group. It can be explained that the intrusion and fouls courseware has a more obvious promotion effect on students mastering the theory of intrusion and fouling. The teaching experiment proves that the way of using multimedia courseware to learn is effective and feasible during the learning process of the students’ offense and foul referee. The rich text, sound, animation and video materials of the multimedia courseware are unified, and the effective editing and application can receive the expected experimental results. Multimedia courseware can visualize the knowledge in the book, the amount of material information is large, the complicated text description is eliminated, the intrusive and foul action can be reproduced, frozen and analyzed, and the teaching situation on the spot is created. In particular, the benign interaction between vision and hearing can better attract and concentrate students' attention and improve learning efficiency. Since the courseware is completed under the condition of "0 class hour" students self-study, it has played a good role in promoting the development of students' self-study ability.

**Table 7:** Intrusion test results

Examination content	Test		Control	
Total score	Average	Variance	Average	Variance
Theoretical knowledge	85.25	6.56	71.42	6.20
Offense judgment	43.73	6.43	36.15	6.24
Examination content	41.52	6.52	35.27	6.81

## Conclusion

The "Wireless Multimedia Communication" course is an important course for information and communication engineering majors. It aims to teach the basic concepts of multimedia technology, multimedia coding, multimedia communication network protocols, multimedia information recovery and reconstruction, multimedia information characterization and communication methods, Multimedia communication systems and other knowledge, and let students apply the knowledge of multimedia communication technology to solve related problems in scientific research and engineering. In the video analysis part, teachers break down actions based on video explanations, students discuss and explore the characteristics of tactical basics, and multimedia courseware control video playback through play, pause, forward and backward, which is convenient for analysis and explanation. Teachers provide test questions, and students conduct test questions to consolidate the mastery of knowledge points. Wireless network multimedia courseware is controlled by programs to automatically change questions, calculate scores, and give standard answers.

The concept of "wireless screen transmission technology" referred to in this article is similar to the above-mentioned scholars. It mainly refers to a wireless network technology that can transmit the content on the client screen to the server screen in real time through wireless transmission and realize real-time display in a wireless network environment. Clients are also source multimedia devices, including portable electronic devices with core control systems such as smart phones and tablet computers. The server refers to the destination multimedia terminal, which refers to a large-screen TV, projector, etc., with good audiovisual effects that are received and displayed in real time.

Algorithms applied in the multimedia field are mainly used to process images. Images are represented by real-number matrices in the program, and image processing ultimately comes down to matrix processing. Although wireless communication applications are complex, the main algorithms can be summarized as the basic linear algebra assembly BLA algorithm, and these algorithms mainly include: matrix multiplication, vector multiplication, matrix addition, norm, convention and other operations. Using software to realize wireless screen transfer technology can be directly pushed through the server and the built-in wireless transmission protocol of the client in combination with the corresponding software, or through some chat tools to assist transmission; the premise of wireless screen transfer through hardware is the client device and the server The devices support the relevant wireless transmission protocol, and then pass the corresponding protocol it is recommended to push to realize wireless screen transmission technology.

## Abbreviations

AVI: Audio video interactive
VCD: Video compact disk
HBCU: Historic black university
WLAN: Wireless local area network
WPAN: Wireless personal area network
WWAN: Wireless wide area network
CDMA: Code division multiple access
GSM: Global system for mobile communications
CPU: Courseware development hardware
